# Sexually Transmitted Infections Detected by Multiplex Real Time PCR in Asymptomatic Women and Association with Cervical Intraepithelial Neoplasia

**DOI:** 10.1055/s-0038-1669994

**Published:** 2018-09

**Authors:** Luiza de Miranda Lima, Carolina René Hoelzle, Renata Toscano Simões, Maria Inês de Miranda Lima, Jordana Rodrigues Barbosa Fradico, Elvis Cristian Cueva Mateo, Danielle Alves Gomes Zauli, Victor Hugo Melo

**Affiliations:** 1 Instituto de Educação e Pesquisa da Santa Casa, Belo Horizonte, MG, Brazil; 2 Research and Development Division, Grupo Hermes Pardini, Vespasiano, MG, Brazil; 3 Faculty of Medicine, Universidade Federal de Minas Gerais, Belo Horizonte, MG, Brazil

**Keywords:** papillomavirus infections, chlamydia, sexually transmitted diseases, polymerase chain reaction, cervical intraepithelial neoplasia, infecções por papilomavírus, clamídia, doenças sexualmente transmissíveis, reação em cadeia de polimerase, neoplasia intraepitelial cervical

## Abstract

**Objective** To determine the frequency of sexually transmitted infections (STIs) in asymptomatic women and the association of STIs with cervical intraepithelial neoplasia (CIN).

**Methods** A cross-sectional study was performed, enrolling women examined in a general gynecology clinic and in a colposcopy referral center from October 2014 to October 2015. The colposcopy group consisted of 71 women, and the general gynecology group consisted of 55 women. Cervical samples were collected for cervical cytology and a multiplex real-time polymerase chain reaction (PCR) was developed to detect human papillomavirus (HPV) and the STIs caused by the following microorganisms: *Chlamydia trachomatis, Mycoplasma hominis, Mycoplasma genitalium, Ureaplasma urealyticum*, and *Neisseria gonorrhoeae*. A multivariate analysis was performed by logistic regression, considering the significance level of 0.05.

**Results** The general frequency of STIs was: 46.8% (HPV); 27.8% (*C. trachomatis*); 28.6% (*M. genitalium*); 0.8% (*M. hominis*); 4.8% (*U. urealyticum*); and 4.8% (*N. gonorrhoeae*). The significant risk factors for CIN were: HPV infection (odds ratio [OR] = 2.53; *p* = 0.024); *C. trachomatis* (OR = 3.04; *p* = 0.009); *M. genitalium* (OR = 2.37; *p* = 0.04); and HPV and *C. trachomatis* coinfection (OR = 3.11; *p* = 0.023). After the multivariate analysis, a significant association was found between HPV and CIN (OR = 2.48; 95% confidence interval [95%CI]: 1.04–5.92; *p* = 0.04); and between *C. trachomatis* and CIN (OR = 2.69; 95%CI: 1.11–6.53; *p* = 0.028).

**Conclusion** The frequency of STIs was high in asymptomatic patients. Infections by HPV and *C. trachomatis* were independently associated with the presence of CIN. The high frequency of STIs in asymptomatic women suggests the need for routine screening of these infections.

## Abstract (pt)

**Resumo:**

**Objetivo** Determinar a frequência de infecções sexualmente transmissíveis (ISTs) em mulheres assintomáticas e a associação destas infecções com a neoplasia intraepitelial cervical (NIC).

**Métodos** Foi realizado um estudo transversal recrutando mulheres atendidas em uma clínica ginecológica geral e em um centro de referência para colposcopia, de outubro de 2014 a outubro de 2015. O grupo de colposcopia consistiu de 71 mulheres, e o grupo de ginecologia geral consistiu de 55 mulheres. Amostras cervicais foram coletadas para citologia cervical e uma reação em cadeia de polimerase (RCP) multiplex em tempo real para detecção do vírus do papiloma humano (HPV) e das ISTs provocadas pelos seguintes micro-organismos: *Chlamydia trachomatis, Mycoplasma hominis, Mycoplasma genitalium, Ureaplasma urealyticum* e *Neisseria gonorrhoeae*. Foi realizada uma análise multivariada por regressão logística, considerando-se o nível de significância de 0,05.

**Resultados** A frequência geral de ISTs foi: 46,8% (HPV); 27,8% (*C. trachomatis*); 28,6% (*M. genitalium*); 0,8% (*M. hominis*); 4,8% (*U. urealyticum*); e 4,8% (*N. gonorrhoeae*). Os fatores de risco significantes para NIC foram: infecção pelo HPV (razão de probabilidades [RP] = 2,53; *p* = 0,024); *C. trachomatis* (RP = 3,04; *p* = 0,009); *M. genitalium* (RP = 2,37; *p* = 0,04); e coinfecção por HPV e *C. trachomatis* (RP = 3,11; *p* = 0,023). Após a análise multivariada, foi encontrada uma associação significante entre HPV e NIC (RP = 2.48; intervalo de confiança de 95% [IC95%]: 1,04–5,92; *p* = 0,04) e entre *C. trachomatis* e NIC (RP = 2,69; IC95%: 1,11–6,53; *p* = 0,028).

**Conclusões** A frequência de ISTs foi alta em mulheres assintomáticas. Infecções por HPV e *C. trachomatis* foram independentemente associadas com a presença de NIC. A alta frequência de ISTs em mulheres assintomáticas sugere a necessidade de rastreamento rotineiro dessas infecções.

## Introduction

Cervical carcinoma is the third most common cancer among women worldwide, and the highest number of cases is being detected in women from developing countries. The carcinogenic human papillomavirus (HPV), a highly prevalent sexually transmitted infection (STI), is a necessary cause to the development of cervical intraepithelial neoplasia (CIN), and it has a central role in the etiology of cervical carcinoma.^1^ However, it has been suggested that other factors may also modulate the risk of the progression of a cervical infection to malignancy.^2^ ^3^ Moreover, only part of the women infected with high-risk HPV will develop CIN/cervical carcinoma, suggesting that the carcinogenic process following HPV infection and CIN are likely influenced by other biological cofactors.^4^

Many studies consider *Chlamydia trachomatis* as a cofactor for the development of CIN, as well as other significant cell abnormalities in women with a history of HPV infection.^2^ ^3^ ^5^ Currently, STIs are a major worldwide public health problem. The most prevalent STIs caused by viruses and bacteria are HPV and *C. trachomatis*. The incidence rate is increasing in developed countries, and according to the World Health Organization (WHO), more than 1 million STIs are contracted every day worldwide.^1^ Untreated genital infection in women may lead to pelvic inflammatory disease, salpingitis, ovarian tube abscess, or perihepatitis, and may present with or without additional complications, such as chronic pelvic pain, ectopic pregnancy, and infertility.^5^

It is estimated that each year 131 million people are infected with *C. trachomatis*, and 78 million people with *Neisseria gonorrhoeae*. The high prevalence of *C. trachomatis* and *N. gonorrhoeae*, including significant rates of asymptomatic infections (50–80% of men and women), and the potential for severe complications are important factors that justify the routine screening of these bacteria.^6^ ^7^

The widespread prevalence of STIs, and the public health costs associated with the difficult diagnosis of these infections have led to the development of fast and reliable methods for diagnosing STIs.^8^ In addition, STIs often go undiagnosed and, when associated with antibiotic resistance, they are also becoming increasingly difficult to treat. Nevertheless, as a great number of pathogens are associated with STIs, and because STIs can be caused by polymicrobial infections, methods to identify multiple pathogens in a single sample are more interesting.^9^ ^10^ Infections such as those caused by *C. trachomatis*, *N. gonorrhoeae*, *Ureaplasma urealyticum*, *Mycoplasma hominis* and *Mycoplasma genitalium* are frequently asymptomatic.^4^ Epidemiological studies on the impact of these infections on HPV contraction and on the development of CIN or cervical carcinoma have yielded equivocal results due to the difficulty in separating biological from behavioral effects.^3^ ^4^

The objective of the present study was to identify the frequency of STIs, associated or not with HPV, and to determine their possible association with CIN among asymptomatic women.

## Methods

### Ethical Aspects

The study was approved by the Ethics in Research Committee of Instituto de Educação e Pesquisa da Santa Casa de Belo Horizonte, Brazil (CAAE-0544.0.000), and registered in the National Ethics in Research Commission (CONEP, in the Portuguese acronym; CAAE-22053313.5.0000.5138).

### Study Population

This is a cross-sectional study that involved 126 women who live in Belo Horizonte, state of Minas Gerais, Brazil, during the period between March 1st, 2014, and September 30th, 2015. The patients were divided into 2 groups: Group 1, composed of 55 women who presented at a general gynecology outpatient clinic (low risk of STI) at Centro de Especialidades Médicas, Santa Casa de Belo Horizonte; and Group 2, composed of 71 women that were referred to a colposcopy clinic (high risk of STI) at Ambulatório de Patologia Cervical of the Centro de Especialidades Médicas, Santa Casa de Belo Horizonte. Among the 71 patients evaluated in the colposcopy clinic, 59 (84%) had abnormal cytology, 41 (59%) underwent a biopsy, 33 (46.5%) presented CIN, and 19 (27%) were CIN 2-3. All patients were asymptomatic for condyloma, abdominal pain, and abnormal vaginal discharge. The exclusion criteria were: pregnancy or postpartum periods, previous hysterectomy, and vaginal bleeding. All of the participants voluntarily agreed to provide a sample for a Pap smear, and for HPV DNA and STIs detection, and signed an informed consent form.

### Clinical Sample Collection

Cervical cytology was performed in all patients. Samples were collected using an Ayre spatula and cytobrush (Cytobrush Kolplast, Ci Ltda, Itapeva, São Paulo, Brazil) for Pap smear collection. The results were reported according to the 2001 Bethesda System.^11^ The scrape samples for the DNA analyses of HPV and STIs were immediately suspended in specimen transport medium and stored at -80°C until the analysis. Women attending the colposcopy referral clinic were submitted to colposcopy and biopsy, when indicated. The CIN diagnosis was considered after the histopathological analysis.

### DNA Extraction

The DNA was extracted from clinical samples as described by Sambrook and Gething.^12^ The concentration and quality of the DNA was determined by spectrophotometry (Nanovue, GE Healthcare, Chicago, IL, US). The DNA extracts were stored at -70°C until used.

### HPV and STI Detection

The detection of HPV was performed by multiplex real-time polymerase chain reaction (M-qPCR) using the general primers GP5 + /GP6+ (de Roda Husman et al)^13^ and PCO3/PCO4 as internal control following the conditions described by Silva et al.^14^

The detection of others STIs was performed by M-qPCR with TaqMan (Applied Biosystems, Foster, CA, US) probes using specific assays to detect simultaneously *N. gonorrhoeae, C. trachomatis, U. urealyticum*, *M. hominis*, and *M. genitalium*. The *β-actin* gene was used as an internal control to ensure the quality of the extracted DNA. Positive STI controls were used in all M-qPCRs, as well as a negative control. All reactions were performed in universal cycling conditions with TaqMan Universal Master Mix buffer in the 7500 Real-Time PCR System (Applied Biosystems, Foster, CA, US). The assays were performed in duplicate for all genes, and the results were analyzed using the 7500 Fast Software, version 2.1 (Applied Biosystems, Foster, CA, US). The samples were analyzed at Instituto de Educação e Pesquisa da Santa Casa de Belo Horizonte, in a partnership with Laboratório Hermes Pardini.

### Statistical Analysis

The two-sided Fisher exact test for the 2 × 2 contingency table was used to evaluate the statistical significance between different groups (general outpatients, colposcopy referral clinic, and patients with or without CIN). Odds ratios (ORs) with 95% confidence intervals (95%CIs) were calculated to estimate the association of HPV, non-HPV, and STIs with CIN. Variables that exhibited statistical significance at the 0.2 level were included in the multivariate analysis using a logistic regression model adjusted by potential confounders. Statistical significance was defined as *p* < 0.05. All statistical analyses were performed using SPSS Statistics for Windows, version 17.0 (SPSS Inc., Chicago, IL, US).

## Results

The characteristics of the patients in each group, such as age, number of partners, smoking habits, early sexual activity, and contraception are shown in Table 1. The mean age of the patients included in the present study was 35 years old. The analyzed variables were not significantly different when the groups were compared.

**Table 1:** Epidemiological characteristics of the patients according to the groups: general outpatient clinic (Group 1) and colposcopy (Group 2)

Characteristics	Group 1 (*n* = 56)	Group 2 (*n* = 71)	Total (*n* = 126)	*p*-value*	OR	95%CI
**Age (years)^#^**						
** < 35**	26 (29.2)	39 (70.8)	65	0.261	1.50	0.73–3.04
** ≥ 35**	30 (21.7)	30 (78.3)	61			
**Number of sexual partners**						
** > 5**	23 (26.3)	34 (73.7)	57	0.401	1.35	0.66–2.75
** < 5**	33 (26.1)	36 (73.9)	69			
**Early sexual activity**						
** < 18**	28 (30.6)	45 (69.4)	73	0.121	1.76	0.85-3.60
** ≥ 18**	28 (20.8)	25 (79.2)	53			
**Smoker**						
** Yes**	9 (25.0)	15 (75.0)	24	0.447	1.42	0.57–3.55
** No**	47 (26.5)	55 (73.5)	102			
**Oral contraception**						
**Yes**	33 (20.3)	36 (79.7)	69	0.401	0.738	0.36-1.50
**No**	23 (33.3)	34 (66.7)	57			

Abbreviations: 95%CI, 95% confidence interval; OR, odds ratio.

Notes: *Two-sided Fisher exact test; ^#^mean age: 35 years old.

The frequency of STIs, independently of CIN occurrence, were: HPV, 46.8%; *M. genitalium*, 28.6%; *C. trachomatis*, 27.8%; *U. urealyticum*, 4.8%; *N. gonorrhoeae*, 4.8%; and *M. hominis*, 0.8%. Table 2 shows the frequency of the STIs among women according to their groups. Infection by HPV was significantly higher among the women in group 2 (colposcopy) (OR = 2.98; 95%CI: 1.43–6.22; *p* = 0.003). No statistical differences between the analyzed groups were observed with the other STIs.

**Table 2:** Frequency of STIs according to general outpatient clinic (group 1) or colposcopy referral center (group 2)

STIs	Group 1 (*n* = 55) %	Group 2 (*n* = 71) %	Total (*n* = 126)	*p*-value*	OR	95%CI
**HPV**						
** Yes**	18 (30.5)	41 (69.5)	59	0.003	2.98	1.43–6.22
** No**	38 (56.7)	29 (43.3)	67			
***Chlamydia trachomatis***						
** Yes**	13 (37.1)	22 (62.9)	35	0.306	1.51	0.68–3.37
** No**	43 (47.3)	48 (52.7)	91			
***Neisseria gonorrhoeae***						
** Yes**	3 (50.0)	3 (50.0)	6	0.779	0.79	0.15–4.07
** No**	53 (44.2)	67 (55.8)	120			
***Mycoplasma genitalium***						
** Yes**	16 (44.4)	20 (55.6)	36	1.000	1.00	0.45–2.17
** No**	40 (44.4)	50 (55.6)	90			
***Mycoplasma hominis***						
** Yes**	01 (100.0)	0 (0)	1	0.444	2.27	1.86–2.77
** No**	55 (44.0)	70 (56.0)	125			
***Ureaplasma urealyticum***						
** Yes**	3 (50.0)	3 (50.0)	6	0.549	0.79	0.15–4.07
** No**	53 (44.2)	57 (55.8)	120			

Abbreviations: 95%CI, 95% confidence interval; HPV, human papillomavirus; STI, sexually transmitted infection; OR, odds ratio.

Notes: *Two-sided Fisher exact test; group 1= general outpatient clinic; group 2= colposcopy referral center.

On the other hand, when comparing the incidence of CIN with STIs, a significant difference was found between the presence of HPV and CIN (OR = 2.53; 95%CI: 1.11–5.75; *p* = 0.024), *C. trachomatis* and CIN (OR = 3.04; 95% CI: 1.30–7.08; *p* = 0.009), and *M. genitalium* and CIN (OR = 2.37; 95%CI: 1.02–5.50; *p* = 0.040). No significant associations were observed with the other STIs (Table 3).

**Table 3:** Frequency of STIs in patients with (+) and without (-) CIN

STIs	CIN + (*n* = 33) %	CIN - (*n* = 93) %	Total (*n* = 126)	*p*-value*	OR	95%IC
**HPV**						
** Yes**	21 (35.6)	38 (64.4)	59	0.024	2.53	1.11–5.75
** No**	12 (17.9)	55 (82.1)	67			
***Chlamydia trachomatis***						
** Yes**	15 (42.9)	20 (57.1)	35	0.009	3.04	1.30–7.08
** No**	18 (19.8)	73 (80.2)	91			
***Neisseria gonorrhoeae***						
** Yes**	01 (16.7)	5 (83.3)	6	0.503	0.55	0.62–4.88
** No**	32 (26.7)	88 (73.3)	120			
***Mycoplasma genitalium***						
** Yes**	14 (38.9)	22 (61.1)	36	0.040	2.37	1.02–5.50
** No**	19 (21.1)	71 (78.9)	90			
***Mycoplasma hominis***						
** Yes**	0 (0.0)	1 (100)	1	0.738	1.35	1.22–1.50
** No**	33 (0.0)	92 (73.6)	125			
***Ureaplasma urealyticum***						
** Yes**	0 (0.0)	6 (100)	6	0.155	1.37	1.23–1.54
** No**	33 (27.5)	87 (72.5)	120			

Abbreviations: 95%CI, 95% confidence interval; CIN, cervical intraepithelial neoplasia; HPV, human papillomavirus; OR, odds ratio; STI, sexually transmitted infection.

Note: *Two-sided Fisher exact test.

Table 4 shows the frequency of HPV and any other STIs associated with the occurrence of CIN. It can be seen that a significant association was found between CIN and HPV plus any other STI, and between CIN and HPV plus *C. Trachomatis*.

**Table 4:** Frequency of coinfection of HPV + STIs associated with the occurrence of cervical intraepithelial neoplasia

Coinfections	CIN + (*n* = 33) %	CIN - (*n* = 93) %	Total	*p*-value*	OR	95%CI
**HPV + STI^#^**						
** Yes**	13 (44.8)	16 (55.2)	29	0.011	3.12	1.29–7.55
** No**	20 (20.6)	77 (79.4)	97			
**HPV + ** ***Chlamydia trachomatis***						
** Yes**	9 (47.4)	10 (52.6)	19	0.023	3.11	1.13–8.53
** No**	24 (22.4)	83 (77.6)	107			
**HPV ** ***+ Mycoplasma genitalium***						
** Yes**	6 (31.6)	13 (68.4)	19	0.562	1.36	0.47–3.95
** No**	27 (25.2)	80 (74.8)	107			

Abbreviations: 95%CI, 95% confidence interval; CIN, cervical intraepithelial neoplasia; HPV, human papillomavirus; OR, odds ratio; STI, sexual transmitted infection.

Notes: *Two-sided Fisher exact test; ^#^ any other STI or more than one*.*

Finally, a multivariate analysis was performed to select the final predictor variables from the univariate model (a *p*-value of 0.15 was considered). A significant association between HPV and CIN (OR = 2.48; 95%CI: 1.04–5.92; *p* = 0.04) and between *C. trachomatis* and CIN (OR = 2.69; 95%CI: 1.11–6.53; *p* = 0.028) was observed.

## Discussion

Cervical cancer remains a leading cause of death among women in developing countries, and HPV has its defined role as an etiological cause of cervical cancer. Human papillomavirus is present in 99.7% of women with uterine cervical cancer, and it is considered the most common sexually transmitted infection worldwide.^3^ However, HPV infections are generally self-limited, and only persistent infections with high-risk HPV are associated with the risk of development of CIN or of invasive cervical cancer. The persistence of the infection has been related to some factors, among them the concomitant infection by *C. trachomatis* and other STIs.^15^ ^16^

The aim of the present study was to evaluate the frequency of STIs in asymptomatic patients, using M-qPCR to identify five genital infections: *C. trachomatis*, *N. gonorrhoeae* and *mollicutes* (*M. genitalium, M. hominis*, and *U. urealyticum*), besides the detection of HPV DNA by conventional PCR.^17^ The frequency of these STIs was detected in two groups of women: one high-risk group (cervical pathology clinic), and a low-risk group (general gynecology outpatient clinic)

Molecular tests by M-qPCR represent the first line option for the diagnosis and screening of STIs in developed countries.^5^ ^6^ ^8^ The advantages of this methodology are not only related to its high specificity and sensitivity, but also to its ability to identify several etiological agents in a single test. The present study used M-qPCR to detect five STIs, but it is possible to make these analyzes for larger numbers of microorganisms. Choe et al,^18^ using M-qPCR for 7 STI agents in 897 urine samples of symptomatic and asymptomatic patients, concluded that the method has high sensitivity and specificity and, above all, enables a rapid simultaneous diagnosis of various STIs.

The present study showed that even in asymptomatic women, belonging or not to a high-risk group, there is a high frequency of STIs, especially HPV (46.8%), *M. genitalium* (28.6%) and *C. trachomatis* (27.8%). Among 126 patients, 66% were infected with at least one STI, and some were simultaneously infected by 2, 3, and even 4 pathogens. In similar investigations, a high prevalence of HPV (46%), *C. trachomatis* (21%), *N. gonorrhoeae* (5%) and *Gardnerella vaginalis* (39%) was reported in symptomatic and asymptomatic groups of women, suggesting that, belonging or not to a high-risk, group, all women should be screened and treated for STIs.^17^ ^19^ In addition, other results are in line with the findings of the present study, reinforcing the need to screen for STIs in asymptomatic women.^20^

Regarding *C. trachomatis,* a larger frequency of infection was found than the one reported in the current literature (27.8%). In general, women from developing countries present a higher prevalence of *C. trachomatis* infection, but not as high as that found in the present study. Some authors have reported prevalence values of *C. trachomatis* ranging from 5 to 22%, reaching 41% among female adolescents.^3^ On the other hand, in developed countries, the prevalence of *C. trachomatis* has presented a frequency ranging from 2 to 10% in the general population, and from 5 to 20% among adolescent females.^21^

Similarly, a high frequency (28.6%) of *M. genitalium* was found, and it was greater than the literature reports.^22^ We would like to point that the sexual transmission and the variable prevalence of *M. genitalium* around the world are important in public health, especially in countries where there is no data on the affected population. Currently, there iis a suggestion to include the routine screening for this STI, considering the possible damage for women's health related to the high prevalence in asymptomatic women. The association of *M. genitalium* with CIN is yet to be established in the literature because this STI has been investigated more recently with the use of PCR for multiple pathogens. At the same time, studies have shown the association of several STIs with CIN without evaluating the isolated association of *M. genitalium* with the cervical neoplasia.^22^ ^23^

*Neisseria gonorrhoeae* was found in 4.8% of the women in the present study, a frequency similar to that reported in the literature, with frequencies ranging from 3 to 12%.^17^ It is important to highlight that all women in the present study had no genital symptoms. In the literature, ∼ 70% of women were asymptomatic, but ∼ 10% of the cases could evolve to salpingitis or pelvic inflammatory disease and infertility.^7^ ^10^

It is known that the presence of HPV is necessary and one of the most important factors for the development of cervical cancer. However, it is also recognized that the role of other different risk factors in association with HPV is crucial in cervical carcinogenesis. Several studies have shown that factors such as smoking, multiparity, prolonged use of contraceptives, and other STIs can triple the risk of development of precancerous lesions, or even of cancer, among women infected with oncogenic types of HPV 16 and 18.^24^ In the present study, HPV types were not investigated.

Epithelial cells play an important role in the induction of an innate immune response in the female genital tract. These cells lined on the mucosal surfaces not only act as a physical barrier, but also actively participate in the secretion of antimicrobial substances and immune factors such as defensins, lactoferrin, and lysozyme. It is believed that an STI can reduce the resolution of HPV infection, with an induction of a pattern of innate and acquired immune response, especially humoral immunity.^25^ In the present study, coinfection by HPV and *C. trachomatis* reached 15% (*p* = 0.023). Some authors have found a high prevalence of coinfection by HPV and *C. trachomatis* in patients with CIN 2-3, suggesting that these infections need to be investigated and treated in young women.^4^ ^26^ The effect of *C. trachomatis* in the cervical epithelium may be defined as a gateway, enabling the HPV to acess the basal layer of the epithelium, increasing the viral load, interacting with HPV high-risk types, and modulating the host immune response by inhibiting the apoptosis process.^25^ ^27^

## Conclusion

Our study demonstrated a high frequency of STIs in asymptomatic women, confirming the association of HPV with CIN, and an unexpected significant association between *C. trachomatis* and CIN. We suggest a discussion about the opportunity of including these STIs in a routine gynecological screening, using biomolecular methods that could detect multiple infections simultaneously, including HPV infection, to avoid the development of CIN.

## References

[1] World Health Organization, Dept. of Reproductive Health and Research. Global Incidence and Prevalence of Selected of Curable Sexually Transmitted Infections 2008 - Geneve, WHO, 2012. http://www.who.int/reproductivehealth/publications/rtis/stisestimates/en/. Accessed January 10, 2017.

[2] Deluca G D Basiletti J Schelover E Chlamydia trachomatis as a probable cofactor in human papillomavirus infection in aboriginal women from northeastern Argentina Braz J Infect Dis 2011 15 06 567 572 Doi: 10.1016/S1413-8670(11)70252-5 22218516 10.1016/s1413-8670(11)70252-5

[3] da Silva Barros N K Costa M C Alves R R Association of HPV infection and Chlamydia trachomatis seropositivity in cases of cervical neoplasia in Midwest Brazil J Med Virol 2012 84 07 1143 1150 Doi: 10.1002/jmv.23312 22585734 10.1002/jmv.23312

[4] Paba P Bonifacio D Di Bonito L Co-expression of HSV2 and Chlamydia trachomatis in HPV-positive cervical cancer and cervical intraepithelial neoplasia lesions is associated with aberrations in key intracellular pathways Intervirology 2008 51 04 230 234 Doi: 10.1159/000156481 18812695 10.1159/000156481

[5] Ashshi A M Batwa S A Kutbi S Y Malibary F A Batwa M Refaat B Prevalence of 7 sexually transmitted organisms by multiplex real-time PCR in Fallopian tube specimens collected from Saudi women with and without ectopic pregnancy BMC Infect Dis 2015 15 569 583 Doi: 10.1186/s12879-015-1313-1 26666587 10.1186/s12879-015-1313-1 PMC4678466

[6] Nassar F A Abu-Elamreen F H Shubair M E Sharif F A Detection of Chlamydia trachomatis and Mycoplasma hominis, genitalium and Ureaplasma urealyticum by polymerase chain reaction in patients with sterile pyuria Adv Med Sci 2008 53 01 80 86 Doi: 10.2478/v10039-008-0020-1 18614434 10.2478/v10039-008-0020-1

[7] Luppi C G de Oliveira R L Veras M A Lippman S A Jones H de Jesus C H Pinho A A Ribeiro M C Caiaffa-Filho H [Early diagnosis and correlations of sexually transmitted infections among women in primarycare health services] Rev Bras Epidemiol 2011 Sep;14 03 467 477 Doi: 10.1590/S1415-790X2011000300011 22069014 10.1590/s1415-790x2011000300011

[8] Greer L Wendel G D Jr Rapid diagnostic methods in sexually transmitted infections Infect Dis Clin North Am 2008 22 04 601 617, v Doi: 10.1016/j.idc.2008.05.010 18954754 10.1016/j.idc.2008.05.010

[9] Samra Z Rosenberg S Madar-Shapiro L Direct simultaneous detection of 6 sexually transmitted pathogens from clinical specimens by multiplex polymerase chain reaction and auto-capillary electrophoresis Diagn Microbiol Infect Dis 2011 70 01 17 21 Doi: 10.1016/j.diagmicrobio.2010.12.001 21392925 10.1016/j.diagmicrobio.2010.12.001

[10] Fredlund H Falk L Jurstrand M Unemo M Molecular genetic methods for diagnosis and characterisation of Chlamydia trachomatis and Neisseria gonorrhoeae: impact on epidemiological surveillance and interventions APMIS 2004 112(11-12):771 784 Doi: 10.1111/j.1600-0463.2004.apm11211-1205.x 15638837 10.1111/j.1600-0463.2004.apm11211-1205.x

[11] Solomon D Davey D Kurman R The 2001 Bethesda System: terminology for reporting results of cervical cytology JAMA 2002 287 16 2114 2119 Doi: 10.1001/jama.287.16.2114 11966386 10.1001/jama.287.16.2114

[12] Sambrook J Gething M J Protein structure. Chaperones, paperones Nature 1989 342(6247):224 225 Doi: 10.1038/342224a0 2572969 10.1038/342224a0

[13] de Roda Husman A M Walboomers J M van den Brule A J Meijer C J Snijders P J The use of general primers GP5 and GP6 elongated at their 3′ ends with adjacent highly conserved sequences improves human papillomavirus detection by PCR J Gen Virol 1995 76(Pt 4):1057 1062 9049358 10.1099/0022-1317-76-4-1057

[14] Silva I D Muniz Y C Sousa M C HLA-G 3'UTR polymorphisms in high grade and invasive cervico-vaginal cancer Hum Immunol 2013 74 04 452 458 Doi: 10.1016/j.humimm.2012.11.025 23228396 10.1016/j.humimm.2012.11.025

[15] Marks M A Gupta S Liaw K L Prevalence and correlates of HPV among women attending family-planning clinics in Thailand BMC Infect Dis 2015 Mar 27;15 159 170 Doi: 10.1186/s12879-015-0886-z 25887797 10.1186/s12879-015-0886-z PMC4387719

[16] Bellaminutti S Seraceni S De Seta F Gheit T Tommasino M Comar M HPV and Chlamydia trachomatis co-detection in young asymptomatic women from high incidence area for cervical cancer J Med Virol 2014 86 11 1920 1925 Doi: 10.1002/jmv.24041 25132162 10.1002/jmv.24041

[17] Rodrigues M M Fernandes P A Haddad J P Frequency of Chlamydia trachomatis, Neisseria gonorrhoeae, Mycoplasma genitalium, Mycoplasma hominis and Ureaplasma species in cervical samples J Obstet Gynaecol 2011 31 03 237 241 Doi: 10.3109/01443615.2010.548880 21417648 10.3109/01443615.2010.548880

[18] Choe H S Lee D S Lee S J Performance of Anyplex™ II multiplex real-time PCR for the diagnosis of seven sexually transmitted infections: comparison with currently available methods Int J Infect Dis 2013 17 12 e1134 e1140 Doi: 10.1016/j.ijid.2013.07.011 24095619 10.1016/j.ijid.2013.07.011

[19] Verteramo R Pierangeli A Mancini E Human Papillomaviruses and genital co-infections in gynaecological outpatients BMC Infect Dis 2009 9 16 23 Doi: 10.1186/1471-2334-9-16 19216747 10.1186/1471-2334-9-16 PMC2656516

[20] Kharsany A B Hoosen A A Moodley J Bagaratee J Gouws E The association between sexually transmitted pathogens and cervical intra-epithelial neoplasia in a developing community Genitourin Med 1993 69 05 357 360 8244352 10.1136/sti.69.5.357 PMC1195117

[21] Fernández-Benítez C Mejuto-López P Otero-Guerra L Margolles-Martins M J Suárez-Leiva P Vazquez F; Chlamydial Primary Care Group. Prevalence of genital Chlamydia trachomatis infection among young men and women in Spain BMC Infect Dis 2013 13 388 396 Doi: 10.1186/1471-2334-13-388 23968487 10.1186/1471-2334-13-388 PMC3765329

[22] Anagrius C Loré B Jensen J S Mycoplasma genitalium: prevalence, clinical significance, and transmission Sex Transm Infect 2005 81 06 458 462 Doi: 10.1136/sti.2004.012062 16326846 10.1136/sti.2004.012062 PMC1745067

[23] Huppert J S Mortensen J E Reed J L Kahn J A Rich K D Hobbs M M Mycoplasma genitalium detected by transcription-mediated amplification is associated with Chlamydia trachomatis in adolescent women Sex Transm Dis 2008 35 03 250 254 Doi: 10.1097/OLQ.0b013e31815abac6 18490867 10.1097/OLQ.0b013e31815abac6 PMC3807598

[24] Franceschi S Herrero R Clifford G M Variations in the age-specific curves of human papillomavirus prevalence in women worldwide Int J Cancer 2006 119 11 2677 2684 16991121 10.1002/ijc.22241

[25] Quayle A J The innate and early immune response to pathogen challenge in the female genital tract and the pivotal role of epithelial cells J Reprod Immunol 2002 57(1-2):61 79 Doi: 10.1016/S0165-0378(02)00019-0 12385834 10.1016/s0165-0378(02)00019-0

[26] Silva J Cerqueira F Medeiros R Chlamydia trachomatis infection: implications for HPV status and cervical cancer Arch Gynecol Obstet 2014 289 04 715 723 Doi: 10.1007/s00404-013-3122-3 24346121 10.1007/s00404-013-3122-3

[27] Fernández G Martró E González V Usefulness of a novel multiplex real-time PCR assay for the diagnosis of sexually-transmitted infections Enferm Infecc Microbiol Clin 2016 34 08 471 476 Doi: 10.1016/j.eimc.2015.10.014 26706392 10.1016/j.eimc.2015.10.014

